# Frequency-Dependent Intrinsic Electrophysiological Functional Architecture of the Human Verbal Language Network

**DOI:** 10.3389/fnint.2020.00027

**Published:** 2020-05-26

**Authors:** Tim Coolen, Vincent Wens, Marc Vander Ghinst, Alison Mary, Mathieu Bourguignon, Gilles Naeije, Philippe Peigneux, Niloufar Sadeghi, Serge Goldman, Xavier De Tiège

**Affiliations:** ^1^Laboratoire de Cartographie fonctionnelle du Cerveau, ULB Neuroscience Institute (UNI), Université libre de Bruxelles (ULB), Brussels, Belgium; ^2^Department of Radiology, CUB—Hôpital Erasme, Université libre de Bruxelles (ULB), Brussels, Belgium; ^3^Magnetoencenphalography Unit, Department of Functional Neuroimaging, Service of Nuclear Medicine, CUB—Hôpital Erasme, Université libre de Bruxelles (ULB), Brussels, Belgium; ^4^Neuropsychology & Functional Neuroimaging Research Unit (UR2NF), Center for Research in Cognition and Neurosciences, ULB-Neuroscience Institute (UNI), Université libre de Bruxelles (ULB), Brussels, Belgium; ^5^BCBL—Basque Center on Cognition, Brain and Language, San Sebastian, Spain; ^6^Laboratoire Cognition Langage et Développement, ULB Neuroscience Institute (UNI), Université libre de Bruxelles (ULB), Brussels, Belgium

**Keywords:** language, magnetoencephalography, rest, brain mapping, nerve net, neuroimaging, humans

## Abstract

Functional magnetic resonance imaging (fMRI) allowed the spatial characterization of the resting-state verbal language network (vLN). While other resting-state networks (RSNs) were matched with their electrophysiological equivalents at rest and could be spectrally defined, such correspondence is lacking for the vLN. This magnetoencephalography (MEG) study aimed at defining the spatio-spectral characteristics of the neuromagnetic intrinsic functional architecture of the vLN. Neuromagnetic activity was recorded at rest in 100 right-handed healthy adults (age range: 18–41 years). Band-limited power envelope correlations were performed within and across frequency bands (θ, α, β, and low γ) from a seed region placed in the left Broca’s area, using static orthogonalization as leakage correction. *K*-means clustering was used to segregate spatio-spectral clusters of resting-state functional connectivity (rsFC). Remarkably, unlike other RSNs, within-frequency long-range rsFC from the left Broca’s area was not driven by one main carrying frequency but was characterized by a specific spatio-spectral pattern segregated along the ventral (predominantly θ and α) and dorsal (β and low-γ bands) vLN streams. In contrast, spatial patterns of cross-frequency vLN functional integration were spectrally more widespread and involved multiple frequency bands. Moreover, the static intrinsic functional architecture of the neuromagnetic human vLN involved clearly left-hemisphere-dominant vLN interactions as well as cross-network interactions with the executive control network and postero-medial nodes of the DMN. Overall, this study highlighted the involvement of multiple modes of within and cross-frequency power envelope couplings at the basis of long-range electrophysiological vLN functional integration. As such, it lays the foundation for future works aimed at understanding the pathophysiology of language-related disorders.

## Introduction

In the last decades, major advances in the understanding of the neurobiology of human verbal language have led to the development of alternatives to the classical Wernicke-Lichtheim-Geschwind model (e.g., Hickok and Poeppel, 2004; Hagoort, 2014; Tremblay and Dick, 2016). The most influential theory (i.e., the *dual stream model*) considers two partially overlapping streams serving different functions: (i) a ventral stream involving bilateral temporal areas for speech-to-meaning processes; and (ii) a left dominant dorsal stream involving fronto-insulo-Wernicke areas for speech-to-articulation mapping (Hickok and Poeppel, 2004, 2007). Additionally, another theory focuses on the central role of Broca’s area for the *Memory, Unification and Control* (MUC) components of language processes. In this model, the left inferior frontal gyrus (IFG) combines lexical items stored in temporo-parietal regions (Memory) into higher-order meaning (Unification) after selection (Control; Hagoort, 2005, 2013, 2014). Rather than narrowing down the neural instantiation of the language function to specific brain nodes or restricted networks, the MUC model considers dynamic, language-relevant interactions between the left IFG and temporo-parietal regions (Hagoort, 2013, 2014). It also highlights the crucial role of interactions between a core fronto-parietal language network and other high-level cognitive networks (such as the attentional or the theory of mind networks; Hagoort, 2014) for successful language processing. This model is in line with the view that language core (i.e., *domain specific*) brain areas in the fronto-temporal regions interact with a vast *domain-general* neural network composed of (non-core) brain regions encompassing, for example, the control, working memory, and salience networks, which may coactivate with the language core depending on task demands (Fedorenko et al., 2013; Fedorenko and Thompson-Schill, 2015; Campbell and Tyler, 2018). Overall, this underlines the critical role of functional integration among language-specific brain areas and between these areas and nodes of other relevant brain networks (e.g., attentional, theory of mind, executive) for integrated and effective verbal language perception, comprehension, and production (Fedorenko et al., 2013; Hagoort, 2014).

The elaboration of those advanced neural models of verbal language function predominantly relied on results from task-related functional magnetic resonance imaging (fMRI) studies (e.g., Vigneau et al., 2006; Price, 2012). However, due to its relatively low temporal resolution, fMRI is unable to give insight into the temporal and spectral dynamics of neural events associated with this major human brain function. Such information could contribute to disentangle the different processes associated with verbal language function *via* their spatial, temporal, and spectral signatures. Indeed, a given brain function may be supported by different neural subnetworks whose oscillatory activities operate at different frequencies (Buzsáki and Draguhn, 2004; Siegel et al., 2012; Lopes da Silva, 2013; Whitman et al., 2013). Time-sensitive electrophysiological techniques, such as magnetoencephalography (MEG), aptly contributed to the characterization of the spatio-temporal spectral dynamics of verbal language processing, which was shown to involve different frequencies (e.g., Goto et al., 2011; Pang and MacDonald, 2012; Lam et al., 2016). Importantly, the frequency of the modulated oscillatory activities was associated with a specific spatial distribution within the verbal language network (vLN; Goto et al., 2011) and also to specific language processes. In a nutshell, variations in activity in the θ and α bands relate to receptive language functions (Bastiaansen et al., 2005; Obleser and Weisz, 2012; Rommers et al., 2017; Wang et al., 2018), such as lexical-semantic retrieval within left temporal areas (Bastiaansen et al., 2005) and language prediction in left fronto-temporal regions (Wang et al., 2018). On the other hand, speech production (Gehrig et al., 2012; Liljeström et al., 2015) and syntactic processes (Ihara et al., 2003) are reflected by changes involving the β and the γ bands in left frontal areas.

Remarkably, even in the absence of any explicit task, i.e., in the so-called *resting state*, brain networks with spatial architectures similar to task-based networks can be recovered using fMRI (e.g., Fox and Raichle, 2007; Raichle, 2010; Deco et al., 2011) or time-sensitive electrophysiological techniques, such as MEG (e.g., de Pasquale et al., 2010; Brookes et al., 2011; Hipp et al., 2012; Wens et al., 2014b; Sjøgård et al., 2019), and electroencephalography (EEG; Siems et al., 2016 ; Liu et al., 2017, 2018; Coquelet et al., 2020). The strong resemblance between the human brain’s intrinsic (i.e., task-free) and extrinsic (i.e., task-evoked) functional architecture suggests that resting-state networks (RSNs) provide a framework for brain responses to the external world (Mennes et al., 2013). RSNs are formed by spatially segregated brain regions bound together by their band-specific power envelopes correlated in time (de Pasquale et al., 2010; Brookes et al., 2011; Hipp et al., 2012; Wens et al., 2014b). Their correlation structure is typically characterized by a main carrying frequency in, for example, the α band [e.g., default-mode network (DMN) and visual RSN] or the β band (e.g., sensorimotor, auditory, and fronto-parietal RSNs). Critically, electrophysiological studies validated the neural basis of the RSNs initially described using fMRI.

Using fMRI, network configurations displaying strong anatomical correspondence to the task-based vLN have been repeatedly extracted at rest (Xiang et al., 2010; Mitchell et al., 2013; Muller and Meyer, 2014; Tie et al., 2014; Branco et al., 2016; Smitha et al., 2017), suggesting the existence of an intrinsic system supporting verbal language functions. To the best of our knowledge, the identification of the electrophysiological, spectrally resolved analog of the fMRI resting-state vLN has not been reported *per se*. Some MEG studies considered resting-state functional connectivity (rsFC) based on (static or dynamic) power envelope correlations among several nodes belonging to six fMRI-based RSNs, including the vLN (de Pasquale et al., 2012; Coquelet et al., 2017). Still, the methodological approaches used in these studies did not fully characterize the spatio-spectral properties of vLN rsFC. Such information would greatly refine the picture of vLN neurophysiology given the aforementioned relationships between spatio-spectral dynamics and functional patterns in the vLN. This would also represent a prerequisite to investigate vLN pathophysiology and functional reorganization phenomena at rest in various brain disorders.

In this MEG study, we investigated the spatio-spectral properties of the vLN intrinsic functional integration using a seed-based rsFC approach, combining within and cross-frequency power envelope correlations, and a *k*-means clustering method in a large resting-state dataset obtained in 100 right-handed healthy adult subjects. Based on previous fMRI studies (Xiang et al., 2010; Muller and Meyer, 2014), we expected to find comprehensive rsFC throughout the vLN using a seed located in Broca’s area given its dual involvement in both the ventral and the dorsal vLN streams, as demonstrated in task-based fMRI (Saur et al., 2008) and its central role in phonological, syntactic, and semantic aspects of verbal language processing (Bookheimer, 2002) in accordance with the MUC model (Hagoort, 2005, 2013, 2014). Based on previous task-based electrophysiological studies (Ihara et al., 2003; Bastiaansen et al., 2005; Goto et al., 2011; Gehrig et al., 2012; Obleser and Weisz, 2012; Pang and MacDonald, 2012; Liljeström et al., 2015; Lam et al., 2016; Rommers et al., 2017; Wang et al., 2018), we predicted that, contrarily to the fMRI vLN that was uncovered as a whole at rest using Broca’s area as a seed region, the neuromagnetic vLN would present a spatial distribution organized into functional subnetworks carried across distinct (within and between) frequency bands.

## Materials and Methods

**Figure 1:**
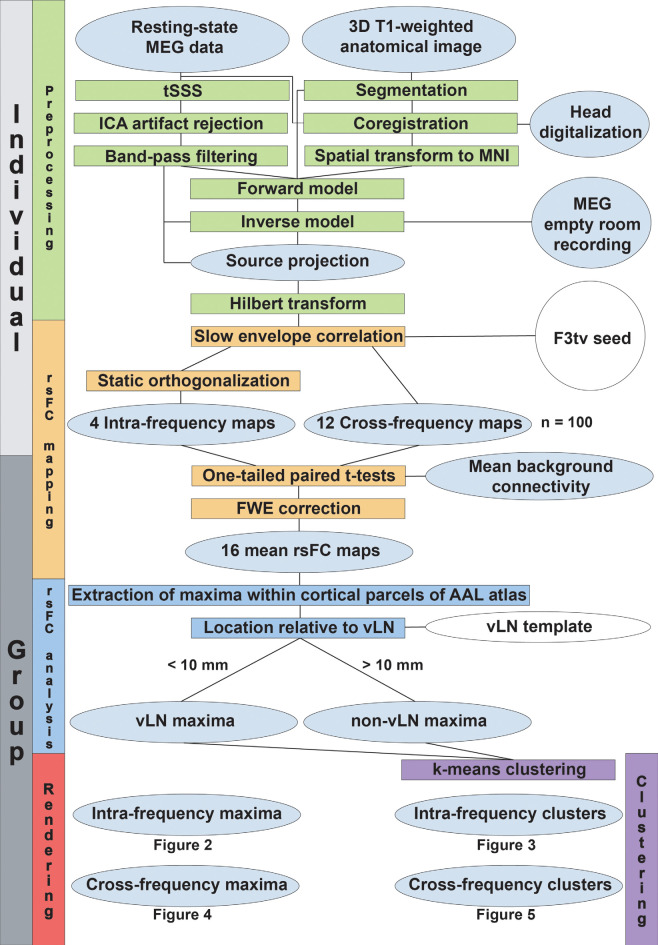
Data processing flowchart. The level of processing (individual or group-level) is indicated in the leftmost boxes. The vertical colored boxes indicate the major processing sections, while their horizontal counterparts detail the processing steps. Gray ellipsoids represent the collected data and the different stages of their transformation. White ellipsoids point to data coming from the literature.

The methods used for MEG signal preprocessing and data analyses are derived from Wens et al. (2014a,b, 2015) and Mary et al. (2017). A flowchart summarizing the data processing steps described in this section is depicted in Figure 1.

### Subjects

One hundred MEG resting-state recordings obtained in right-handed healthy adults (mean age: 26.5 years, range: 18–41 years; 48 females) were used in this study. Resting-state data were collected in the course of different MEG studies (Bourguignon et al., 2011, 2013; Clumeck et al., 2014; Marty et al., 2015; Mary et al., 2015; Vander Ghinst et al., 2016). None of the subjects had a history of neurologic or psychiatric disease. Handedness was assessed by the Edinburgh Handedness Inventory scale (Oldfield, 1971). All studies were approved by the CUB—Hôpital Erasme Ethics Committee, and informed consent was obtained from each subject.

### Data Acquisition and Preprocessing

MEG data were recorded (band-pass: 0.1–330 Hz, sampling rate: 1 kHz) in a lightweight magnetically shielded room (MSR; Maxshield™, MEGIN, Helsinki, Finland) using a 306-channel whole-scalp-covering neuromagnetometer (Neuromag Vectorview™, MEGIN, Helsinki, Finland). Characteristics of the MSR and MEG system were previously described (De Tiège et al., 2008). During resting-state recordings, subjects were asked to sit still with their head in the MEG helmet while gazing at a fixation cross on a screen or a point on the opposite wall of the MSR for 5 min. Four head-tracking coils monitored the subjects’ head position inside the MEG helmet. The locations of the coils and at least 150 head-surface points (on the scalp, nose, and face) with respect to anatomical fiducials were recorded with an electromagnetic tracker (Fastrak, Polhemus, Colchester, VT, USA).

Subjects’ high-resolution 3D-T1 weighted magnetic resonance image (MRI) were also acquired using a 1.5 T MRI scanner (Intera, Philips, Netherlands) after the MEG recordings.

The raw MEG data were preprocessed off-line using the temporal extension of the signal space separation method (Taulu et al., 2005) to reduce external interferences and correct for head movements (MaxFilter™ v2.2 with default parameters, MEGIN, Helsinki, Finland). Cardiac, eye-movement, and electronic artifacts were identified by independent component analysis (FastICA algorithm with rank reduction to 30 and nonlinearity *tanh*; Hyvärinen and Oja, 2000; RRID:SCR_013110) applied to sensor time series filtered between 0.5 and 45 Hz (Vigário et al., 2000) and visual inspection of the components (number of identified components per subject: 5.0 ± 1.4, mean ± SD). Artifactual components were then regressed out from the full-rank data (without the 0.5–45 Hz filter). The resulting signals were finally filtered in exclusive, non-overlapping language-related frequency bands identified by Goto et al. (2011): θ (5–8 Hz), α (8–13 Hz), β (13–25 Hz), and low γ (25–45 Hz) bands. Both pre- and post-ICA filters consisted in basic Fourier-transform filters whereby spectral coefficients outside the band of interest are set to zero. The initial and the last second of filtered data were removed to avoid boundary effects.

### Source Reconstruction

Individual anatomical MRIs were segmented using Freesurfer software (Fischl, 2012; Martinos Center for Biomedical Imaging, MA, USA; RRID:SCR_001847). The MEG and MRI coordinate systems were co-registered using the three anatomical fiducial points for initial estimation and the head-surface points to manually refine the surface co-registration. Individual MEG forward models were then computed using the single-layer Boundary Element Method implemented in the MNE-C software suite (Gramfort et al., 2014, Martinos Center for Biomedical Imaging, MA, USA; RRID:SCR_005972). To ease the group-level analysis, forward models were based on a source grid obtained from a common 5-mm cubic grid containing 16,102 source locations and built in the Montreal Neurological Institute (MNI) template brain by applying a non-linear spatial deformation algorithm implemented in Statistical Parametric Mapping (SPM8, Wellcome Department of Cognitive Neurology, London, UK; RRID:SCR_007037).

The resulting forward models were then inverted *via* band-specific Minimum Norm Estimation (MNE; Dale and Sereno, 1993) using gradiometer signals only (Garcés et al., 2017). For each frequency band, sensor-space noise covariance was estimated from 5 min of artifact-free data recorded from an empty room and filtered both spatially using signal space separation and temporally in the relevant frequency bands. The MNE regularization parameter was fixed according to the band-specific MEG signal-to-noise ratio (which varies across the frequency spectrum; Hari and Puce, 2017) *via* the prior consistency condition (Wens et al., 2015). The dimension of each dipole moment was reduced from 3 to 1 by projection onto the direction of maximum variance. Finally, the analytic signal was derived using the Hilbert transform.

### Seed-Based Functional Connectivity Mapping

To create maps of seed-based rsFC, we used slow envelope correlation, whereby the Pearson temporal correlation is estimated between the seed’s and all other target sources’ slow envelope signals (i.e., low-pass Fourier-transform filtered at 1 Hz) since this allows for better identification of RSN couplings (see e.g., Hipp et al., 2012; Hall et al., 2014). Within-frequency rsFC relied on seed and target activity in the same frequency band and was preceded by static orthogonalization of the target sources with respect to the seed for spatial leakage correction (Brookes et al., 2012), which is moderately conservative and relatively resilient to seed mislocation (Wens et al., 2015). For cross-frequency rsFC, the frequency band of the seed and targets were different. In this case, no leakage correction was necessary since MNE spatial leakage is band-independent (Wens et al., 2015; Wens, 2015), and, furthermore, two signals filtered in non-overlapping frequency bands are inherently orthogonal.

For each pair of frequency bands, the individual seed-based rsFC maps were used to build a statistical *t* map disclosing the regional rsFC patterns exceeding the background rsFC, i.e., the mean correlation value over the whole brain. This allowed us to identify RSNs as regions of significant regional rsFC peaks (de Pasquale et al., 2010). Of note, this technique would be *a priori* insensitive to negative regional rsFC; however, the only known case of RSN anticorrelation identified with fMRI (Fox et al., 2005) has not been disclosed with MEG (see de Pasquale et al., 2010; for a discussion Wens et al., 2019). Significance was assessed using one-tailed, paired *t* tests at *p* < 0.05 corrected for the multiple comparisons inherent to mass-univariate statistical testing across the whole brain volume (16,102 source locations). The family-wise error rate was controlled by Bonferroni correction for the effective number *ρ* of spatial degrees of freedom in MNE maps, which is much lower than 16,102 due to spatial blurriness of neuromagnetic fields and can be estimated from the rank of the MEG forward model (Wens et al., 2015). Here, we obtained *ρ* = 58, corresponding to an uncorrected significance level of *p* < 0.05/*ρ* = 8.6 × 10^−3^. This approach provides an electrophysiological analog of random field theory often applied in fMRI (Brett et al., 2003).

A single seed location was chosen in the left IFG to disclose the vLN in view of its involvement in both language streams (Saur et al., 2008). The MNI coordinates of the seed [i.e., (–43, 20, 4) mm] were determined as the center of mass of an activation cluster defined in a large meta-analysis (Vigneau et al., 2006) in the ventral part of the pars triangularis (F3tv). This particular location was chosen because of its central and sulcal position in the left IFG so as to optimally capture the signal of a tangential source that is not contaminated by an adjacent gyrus (i.e., precentral or middle frontal gyrus). Functionally, it lies within Brodmann’s area 45, which appears to be particularly selective to language processing compared to left IFG domain-general functions, such as hierarchical structure building, action processing, working memory, or cognitive control (Fedorenko et al., 2012). Furthermore, its connectivity profile encompasses phonological, syntactic, and semantic regions of the vLN previously demonstrated in fMRI (Xiang et al., 2010). Besides, the choice of a seed in Broca’s rather than Wernicke’s area was favored due to the looser anatomical definition of the latter brain region (Bogen and Bogen, 1974; Binder, 2015). It was further motivated by previous seed-based resting state fMRI studies that demonstrated significant functional connectivity with the whole vLN from a seed placed within Broca’s area (Xiang et al., 2010; Muller and Meyer, 2014).

For each *t*-map of rsFC, we extracted the value and MNI coordinates of all supra-threshold local maxima. We here focused on local maxima because they are indicative of genuine rsFC rather than field spread. Notwithstanding, even though we used appropriate spatial leakage correction, topographical patterns of rsFC are generally fraught with spurious correlation related to “secondary” spatial leakage effects (Wens et al., 2015; Palva et al., 2018). For this reason, we refrained from interpreting the absolute value of the connectivity measure. Maxima falling outside the supratentorial cortical parcels of the Automated Anatomical Labelling atlas (AAL; Tzourio-Mazoyer et al., 2002; RRID:SCR_003550) were discarded as artifactual deep activity. The resulting connections were visualized on a surface-rendered MNI brain template using the BrainNet Viewer available at http://www.nitrc.org/projects/bnv (Xia et al., 2013; RRID:SCR_009446).

### Relevance to the Verbal Language Network

In order to evaluate the spatial relationship of rsFC maxima to the vLN as disclosed by fMRI, we built a vLN template using the Neurosynth online platform (Yarkoni et al., 2011; RRID:SCR_006798). The vLN template was created from the recommended *association test* map (*p* < 0.01, corrected for False Discovery Rate), obtained after performing a large-scale automated meta-analysis using the term *language*, which included 885 fMRI studies (technical details available online at http://neurosynth.org). A local rsFC maximum was deemed to fall within the vLN template when its distance to the mask (i.e., the Euclidean distance to the closest in-mask voxel) was less than 10 mm. This accounted for the stringent limitation of using only focal rsFC maxima, the lesser spatial precision of MEG compared to fMRI, and the mean distance difference of the order of the centimeter in studies directly comparing the spatial location of fMRI vs. MEG activation centroids (e.g., Stippich et al., 1998; Schulz et al., 2004; Liljeström et al., 2009).

### Spatial Clustering

To test the working hypothesis that the neuromagnetic resting-state vLN is characterized by spatio-spectrally segregated intra- and cross-frequency subnetworks, we classified all the F3tv-based band-specific connections into clusters on the basis of the MNI coordinates of corresponding local maxima. This also minimized irrelevant localization displacements (e.g., the same connection involved in different frequency bands may appear at nearby but distinct coordinates). Crucially, the classification gathers all local maxima across all considered frequency band pairs but is not informed by them, which enables the testing of our hypothesis. In practice, we used *k*-means clustering (Lloyd, 1982) with Euclidean distance as the clusterization index to partition the *n* rsFC local maxima into *k* clusters of the smallest size, represented by the within-cluster sum of squares (SSW), while keeping the complexity of the classification (i.e., the number *k* of clusters) at the lowest, hence avoiding overfitting. This compromise was determined with *elbow criterion*. Specifically, we set this parameter automatically by running the *k*-means clustering for all possible values of *k* (1 ≤ *k* ≤ *n*) and determining the point on the resulting elbow curve (i.e., SSW as a function of *k*) where its tangent is most parallel to the chord joining the first and last points.

## Results

### Within-Frequency Electrophysiological Resting-State Functional Integration From the Left Inferior Frontal Gyrus

**Figure 2:**
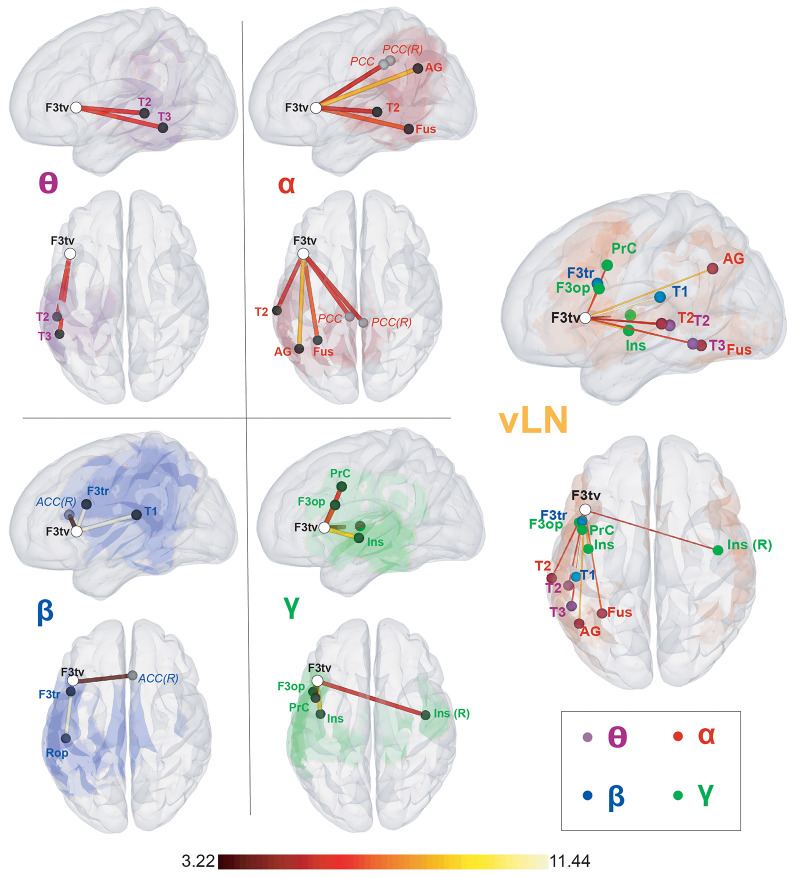
Seed-based within-frequency resting-state functional connectivity (rsFC) maps and location of their local resting-state functional connectivity (rsFC) maxima. Left sagittal and top surface rendering pairs. The seed (F3tv) location is indicated by a white disc. In the left part of the figure, the first four pairs depict for each frequency band (θ, α, β, and low γ) the rsFC maxima belonging to the verbal language network (vLN) (black discs) and those outside the vLN mask (gray discs), superimposed on the corresponding rsFC statistical map (thresholded at *t* > 3.22; *p* < 0.05, corrected for the family-wise error rate). The rightmost pair gathers all rsFC maxima within the vLN, superimposed on the vLN mask (orange background), with nodes colored according to their frequency band (θ: purple, α: red, β: blue, low γ: green). The color of the edges connecting the seed and the rsFC maxima codes for the rsFC *t-values* for which a scale is provided at the bottom. Anatomical abbreviations are listed in the legend of Table 1.

Table 1 (diagonal) and Figure 2 detail the location of rsFC local maxima obtained within each frequency band.

**Table 1:** Location of rsFC local maxima for each pair of frequency bands.

		Frequency band of rsFC maxima															
		θ				α				β				Low γ			
		MNI			Loc	MNI			Loc	MNI			Loc	MNI			Loc
**Frequency band of seed F3tv**	θ	**−54**	**−32**	**−1**	**T2**	−43	13	3	Ins	−35	10	10	Ins	−50	−59	35	AG
		**−52**	**−46**	**−12**	**T3**	−41	−3	−4	Ins	−55	−50	28	SMG	−22	−64	7	Cal
						−41	15	17	F3op	−54	−30	12	T1	-61	−35	40	IPL
						−52	−41	28	SMG					−60	−34	42	IPL
						−55	−8	7	T1					−57	−19	51	IPL
						47	−38	16	T1(R)					*46*	−*35*	*63*	IPL(R)*
														−*19*	−*53*	*61*	PC*
														*12*	−*62*	*60*	PC(R)*
														−55	−41	25	SMG
	α	−40	−60	44	AG	**−47**	**−58**	**34**	**AG**	*11*	−*45*	*29*	PCC(R)*	−51	−28	44	IPL
		−33	−64	47	AG	**−32**	**−51**	**−13**	**Fus**	−67	−32	11	T1	−*15*	−*58*	*55*	PC*
		*40*	−*57*	*26*	AG(R)*	**−6**	**−32**	**37**	PCC*	−68	−33	9	T2	*7*	−*58*	*15*	PC*
		*32*	−*53*	−*19*	Fus(R)^*^	**6**	**−37**	**40**	PCC(R)*					*7*	−*62*	*49*	PC(R)*
		−*2*	−*69*	*48*	PC*	**−65**	**−27**	**1**	**T2**
		−64	−27	18	SMG												
		*21*	−*76*	*31*	SOG(R)*												
		−65	−28	15	T1												
		−69	−35	5	T2												
		−51	−61	20	T2												
		−43	−64	10	T2												
	β	−28	−50	44	IPL	−44	6	15	F3op	**6**	**26**	**6**	ACC(R)*	*5*	−*30*	*41*	PCC(R)*
		−58	−23	18	SMG	−46	−50	26	SMG	**−45**	**13**	**25**	**F3tr**	−50	−37	31	SMG
		−46	−54	18	T2						**−49**	**−26**	**17**	T1			
		−50	−47	−12	T3					
	**Low** γ	No supra-threshold voxels				−49	−1	38	PrC	−55	14	18	F3op	**−41**	**−7**	**−4**	**Ins**
										−*21*	−*38*	−*4*	PH^*^	**−47**	**12**	**22**	**F3op**
														**−45**	**7**	**36**	**PrC**
														**−45**	**−8**	**6**	**Ins(R)**

Within-frequency rsFC maxima are located on the diagonal (bold font). MNI coordinates are given for each maximum (in mm) along with their anatomical location (Loc). All maxima lie within the left hemisphere unless specified otherwise, i.e., (R): right hemisphere, and belong to the vLN, except those italicized and marked with an asterisk (*). Abbreviations: ACC, Anterior cingulate cortex; AG, Angular gyrus; Cal, Calcarine cortex; Ins, Insula; IPL, Inferior parietal lobule; PC, Precuneus; SMG, Supramarginal gyrus; SOG, Superior occipital gyrus; T1, Superior temporal gyrus; F3op, Pars opercularis (inf. frontal gyrus); F3tr, Pars triangularis (inf. frontal gyrus); Fus, Fusiform gyrus; PCC, Posterior cingulate cortex; PH, Parahippocampal gyrus; PrC, Precentral gyrus; T2, Middle temporal gyrus; T3, Inferior temporal gyrus.

The F3tv seed established within-frequency long-range rsFC with a total of *n* = 14 rsFC maxima across the four frequency bands. Among those, 11 were included in the vLN template. Two vLN maxima were identified in the θ band, three in the α band, two in the β band, and four in the low γ band. Ten out of the 11 vLN rsFC maxima were located in the left hemisphere, and only one was located in the right hemisphere. Three rsFC maxima fell out of the fMRI-based vLN template and were located in the posterior cingulate cortex (PCC) bilaterally in the α band and in the right anterior cingulate cortex (ACC) in the β band.

### Spatial Clustering of Within-Frequency Functional Connectivity Maxima

**Figure 3:**
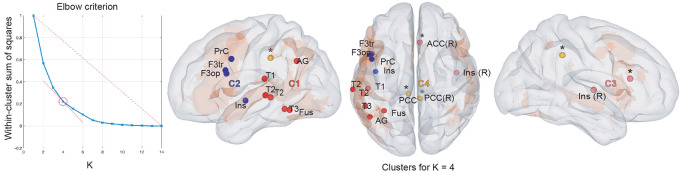
Results of within-frequency spatial clustering. First column: elbow criterion definition. Elbow curve and parameter selection. Second to fourth columns: spatial clustering of within-frequency rsFC local maxima across all frequency bands for *K* = 4. Left sagittal, top and right sagittal surface brain renderings with clusters 1 (red), 2 (blue), 3 (pink), and 4 (yellow disks). All maxima belong to the vLN (orange background), except those marked with an asterisk (*). Anatomical abbreviations are listed in the legend of Table 1.

Table 2 and Figure 3 summarize the results of the spatial clustering of within-frequency rsFC maxima.

**Table 2:** Results of spatial clustering of within-frequency rsFC local maxima.

C	B	MNI			Loc
**1**	**θ**	−54	−32	−1	T2
	**θ**	−52	−46	−12	T3
	**α**	−47	−58	34	AG
	**α**	−32	−51	−13	Fus
	**α**	−65	−27	1	T2
	**β**	−49	−26	17	T1
**2**	**β**	−45	13	25	F3tr
	**Low γ**	−47	12	22	F3op
	**Low γ**	−41	−7	−4	Ins
	**Low γ**	−45	7	36	PrC
**3**	**β**	*6*	*26*	*17*	ACC(R)*
	**Low γ**	45	−8	6	Ins(R)
**4**	**α**	−*6*	−*32*	*37*	PCC*
	**α**	*6*	−*37*	*40*	PCC(R)*

The first column indicates the cluster number (**C**). Within each cluster, the second column represents the frequency band (**B**) corresponding to each maximum. Their MNI coordinates are also given (in mm) along with their anatomical location (**Loc**). All maxima lie within the left hemisphere unless specified otherwise, i.e., (R): right hemisphere, and belong to the vLN, except those italicized and marked with an asterisk (*). Anatomical abbreviations are listed in the legend of Table 1.

Based on the elbow criterion, we considered the case of *k* = 4 clusters to partition our *n* = 14 within-frequency rsFC local maxima (see Figure 3, left). The two largest clusters regrouped rsFC local maxima that all belonged to the vLN and the left hemisphere. The first was a temporo-parietal cluster (see cluster 1 in Figure 3, right) comprising six rsFC local maxima mainly in the θ and α frequency bands. The second was a fronto-insular cluster with four rsFC local maxima in the β and low γ frequency bands (cluster 2). A third smaller cluster included two right-sided rsFC local maxima in the β and low γ frequency bands; one insular in the vLN and another in the ACC, outside the vLN (cluster 3). The fourth and equally small cluster was defined by the two bilateral PCC rsFC local maxima in the α frequency band, both falling outside the vLN (cluster 4).

### Cross-Frequency Electrophysiological Resting-State Functional Integration From the Left Inferior Frontal Gyrus

**Figure 4:**
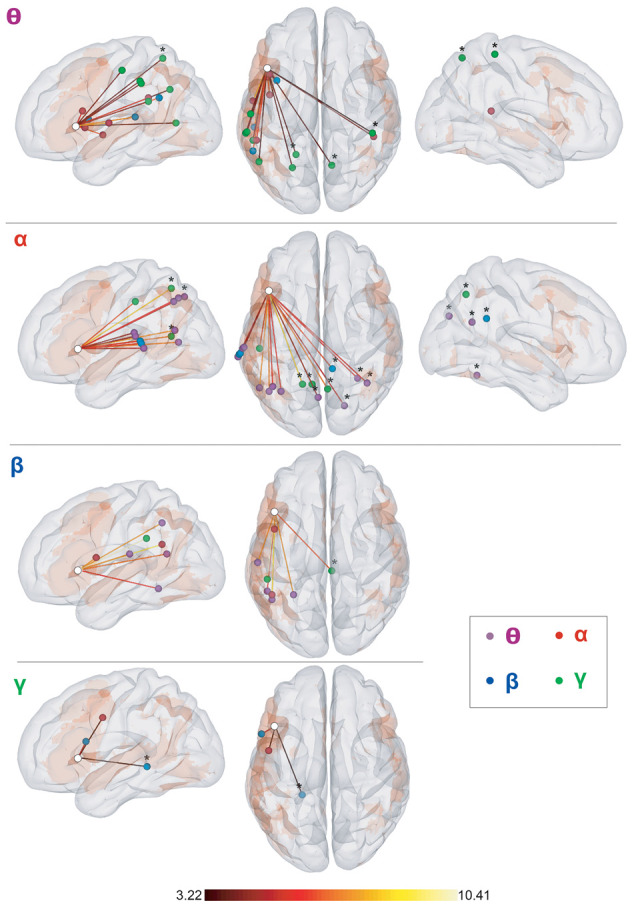
Seed-based cross-frequency rsFC local maxima. Left sagittal, top and right sagittal surface renderings. The seed (F3tv) location is indicated by a white disc. Each row represents the frequency band (θ, α, β, and low γ) of the seed and depicts the supra-threshold cross-frequency rsFC maxima across the other bands. The color of the disk indicates the frequency band of the maxima (θ: purple, α: red, β: blue, low γ: green). All maxima belong to the vLN (orange background), except those marked with an asterisk (*). The color of the edges connecting the seed and the rsFC maxima codes for the rsFC *t*-values for which a scale is provided at the bottom. Anatomical annotations are not indicated on the maxima due to the large number of maxima but are listed in Table 1 off-diagonal.

Table 1 (off-diagonal) and Figure 4 identify the location of cross-frequency rsFC local maxima.

The twelve cross-frequency long-range rFC of the F3tv seed obtained for each combination of distinct frequency bands gave rise to a total of *n* = 47 rsFC maxima, of which 34 belonged to the vLN. Thirty-eight rsFC maxima were located in the left hemisphere, including 33 attributed the vLN. Among the nine remaining maxima in the right hemisphere, eight were outside of the vLN.

Of note, the number of rsFC maxima was larger for the interactions with F3tv in the lower frequency bands (θ: 18 maxima, α: 18, β: 8, and low γ: 3).

### Spatial Clustering of Cross-Frequency Functional Connectivity Maxima

**Figure 5:**
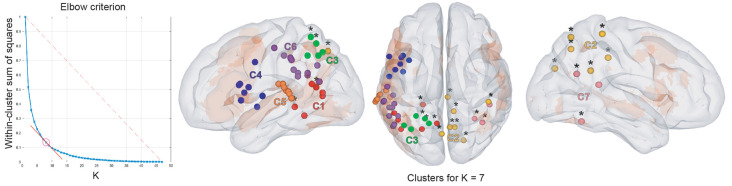
Results of cross-frequency spatial clustering. Leftmost column: elbow criterion definition. Elbow curve and parameter selection. Second to fourth columns: Spatial clustering of cross-frequency rsFC local maxima across all frequency bands for *K* = 7. Left, top, and right sagittal surface brain renderings with clusters 1 (red), 2 (yellow), 3 (green), 4 (blue), 5 (orange), 6 (purple), and 7 (pink disks). All maxima belong to the vLN (orange background), except those marked with an asterisk (*). Anatomical annotations are not indicated on the maxima due to their large number but are listed in Table 3.

Table 3 and Figure 5 summarize the results of the spatial clustering of cross-frequency rsFC local maxima.

**Table 3:** Results of spatial clustering of cross-frequency rsFC local maxima.

C	B_seed_	B_max_	MNI			Loc
**1**	**θ**	**Low γ**	−22	−64	7	Cal
	**α**	**θ**	−43	−64	10	T2
		**Low γ**	−*7*	−*58*	*15*	PC*
	**β**	**θ**	−46	−54	18	T2
			−50	−47	−12	T3
	**Low γ**	**β**	−*21*	−*38*	−*4*	PH*
**2**	**θ**	**Low γ**	*46*	−*35*	*63*	IPL(R*)
			*12*	−*62*	*60*	PC(R)*
	**α**	**θ**	−*2*	−*69*	*48*	PC*
			*21*	−*76*	*31*	SOG(R)*
		**β**	*11*	−*45*	*29*	PCC(R)*
		**Low γ**	*7*	−*62*	*49*	PC(R)*
	**β**	**Low γ**	*5*	−*30*	*41*	PCC(R)*
**3**	**θ**	**Low γ**	−19	−53	61	PC*
	**α**	**θ**	−40	−60	44	AG
			−33	−64	47	AG
		**Low γ**	−*15*	−*58*	*55*	PC*
	**β**	**θ**	−28	−50	44	IPL
**4**	**θ**	**α**	−41	15	17	F3op
			−43	13	3	Ins
			−41	−3	−4	Ins
			−55	−8	7	T1
		**β**	−35	10	10	Ins
	**β**	**α**	−44	6	15	F3op
	**Low γ**	**α**	−49	−1	38	PrC
		**β**	−55	14	18	F3op
**5**	**θ**	**β**	−54	−30	12	T1
	**α**	**θ**	−64	−27	18	SMG
			−65	−28	15	T1
			−69	−35	5	T2
		**β**	−67	−32	11	T1
			−68	−33	9	T2
	**β**	**θ**	−58	−23	18	SMG
**6**	**θ**	**α**	−52	−41	28	SMG
		**β**	−55	−50	28	SMG
		**Low γ**	−50	−59	35	AG
			−61	−35	40	IPL
			−60	−34	42	IPL
			−57	−19	51	IPL
			−55	−41	25	SMG
	**α**	**θ**	−51	−61	20	T2
		**Low γ**	−51	−28	44	IPL
	**β**	**α**	−46	−50	26	SMG
		**Low γ**	−50	−37	31	SMG
**7**	**θ**	**α**	47	−38	16	T1(R)
	**α**	**θ**	*32*	−*53*	−*19*	Fus(R)*
			*40*	−*57*	*26*	AG(R)*

The first column indicates the cluster number (**C**). Within each cluster, the second column represents the frequency band of the seed F3tv (**B_seed_**) while the third column shows the band of the rsFC maxima (**B_max_**). MNI coordinates are also given for each maximum (in mm) along with their anatomical location (**Loc**). All maxima lie within the left hemisphere unless specified otherwise, i.e., (R): right hemisphere, and belong to the vLN, except those italicized and marked with an asterisk (*). Anatomical abbreviations are listed in the legend of Table 1.

Based on the elbow criterion, we considered the case of *k* = 7 clusters to partition our *n* = 47 cross-frequency rsFC local maxima (see Figure 5, left). Most clusters were composed of rsFC local maxima that were involved in cross-frequency couplings encompassing multiple frequency bands.

Clusters 4, 5, and 6 (Figure 5, right) were exclusively composed of left hemisphere vLN maxima, spanning over dorso-rostral fronto-temporo-insular regions (cluster 4) as well as postero-superior temporal and inferior parietal areas (clusters 5 and 6). These three clusters amounted to 26 maxima. Clusters 1 and 3 also comprised left posterior temporal (cluster 1) and left inferior parietal (cluster 3) regions within the vLN as well as maxima outside of the vLN in the left precuneus. Of note, a calcarine maximum within the vLN and a parahippocampal maximum outside of the vLN were included in cluster 1. Cluster 2 was solely composed of non-vLN maxima, distributed over the bilateral posterior parasagittal regions (bilateral precuneus and right posterior cingular cortex) and right inferior parietal lobule (IPL). Finally, cluster 7 involved three maxima in the right temporo-parietal region, one of which was deemed to belong to the vLN in the superior and posterior aspect of the superior temporal gyrus, restricted to cross-frequency couplings involving lower frequency bands (i.e., θ and α).

## Discussion

This MEG study relied on band-specific power envelope correlations based on a core language seed in the left IFG (F3tv) to reconstruct the electrophysiological static vLN in a large population of 100 right-handed healthy adult subjects.

The main original findings discussed hereafter are that spatial patterns of long-range, within-frequency rsFC from the left IFG (F3tv) seed: (i) are frequency-band dependent and not restricted to a single band contrarily to other neuromagnetic RSNs; (ii) identify intrinsic functional integration of Broca’s area along the ventral vLN stream in low frequency bands (predominantly θ and α) and the dorsal vLN stream in higher frequency bands (β and low γ); (iii) are dominated by left hemisphere functional integration; and (iv) involve cross-network interactions with nodes of the executive control network and of the DMN.

Similarly, spatial patterns of long-range, cross-frequency rsFC from this seed are also dominated by left hemisphere functional integration and identify a cross-frequency intrinsic neuromagnetic vLN that is also characterized by a distinction between the ventral and dorsal vLN streams in the left hemisphere. However, contrary to within-frequency rsFC, this spatial segregation lacked a clear seed-to-target frequency band organization, potentially reflecting large-scale functional integration over multiple spatial and temporal scales within the vLN. Finally, cross-network interactions with nodes of the DMN are also characterized by cross-frequency functional integration.

### Band-Specific Within-Frequency Long-Range Intrinsic Functional Connectivity in the vLN

This study demonstrates that the rsFC within the neuromagnetic vLN cannot be subsumed by a main carrying frequency even when limited to classical within-frequency coupling. This is a stark contradistinction with other neuromagnetic RSNs initially uncovered using fMRI (de Pasquale et al., 2010; Brookes et al., 2011; Hipp et al., 2012; Wens et al., 2014b). In fact, the spectral content of neuromagnetic RSNs broadly coincides with the classical θ, α, β, and low-γ bands (Vidaurre et al., 2018). Here, different frequency bands ranging from θ to low γ were necessary to map the neuromagnetic resting-state vLN along the left perisylvian (temporal: θ and α; parietal: α and β; fronto-insular: β and low-γ) regions of the fMRI-based vLN template.

This finding sheds light on the spectral components of the rsFC constituting the resting state vLN that was previously uncovered by fMRI studies using left IFG seeds (Xiang et al., 2010; Muller and Meyer, 2014) or data-driven approaches (e.g., Mitchell et al., 2013; Branco et al., 2016). We here show that the within-frequency part of the neuromagnetic vLN is actually spatially segregated based on spectral characteristics, which may relate to the different functional roles of the left IFG.

### Spatio-Spectral Signature of the Within-Frequency Functional Integration of Broca’s Area Within the vLN

The frequency-dependent spatial distribution of the within-frequency functional integration from our seed (F3tv) in Broca’s area was confirmed by the spatial clustering analysis. It identified two main left hemisphere vLN clusters driven by different frequency bands and segregated along a ventro-dorsal and caudo-rostral axis. Local maxima of correlation were indeed located more dorsal and rostral in brain regions as the frequency increased, and the pattern was similar to task-elicited activations observed in Goto et al. (2011). In light of the dual stream (Hickok and Poeppel, 2004, 2007) and the MUC (Hagoort, 2005, 2013, 2014) models of speech processing, we propose that this spatio-spectral pattern of within-frequency electrophysiological rsFC from Broca’s area is of functional relevance.

In keeping with the role historically given to Broca’s area, the dorso-rostral cluster (within-frequency cluster 2 in Figure 3) involved β and low-γ frequency bands, which were previously implicated in speech production (Gehrig et al., 2012; Liljeström et al., 2015) and syntax processing (Ihara et al., 2003). It encompassed left fronto-insular regions attributed to the dorsal stream (Hickok and Poeppel, 2007), including the pars opercularis of the IFG and the dorsal premotor cortex, that have been linked to syntactic and phonological unification processes (Hagoort, 2013).

On the other hand, the ventro-caudal cluster (within-frequency cluster 1 in Figure 3) involved mainly the θ and α frequency bands and, to a lesser extent, the β frequency band, which were previously linked to language comprehension (Bastiaansen et al., 2005; Obleser and Weisz, 2012) and the representation of sentence-level meaning (Lewis et al., 2016; Rommers et al., 2017; Wang et al., 2018). It incorporated posterior temporal regions of the ventral stream (Hickok and Poeppel, 2007), extending to the angular gyrus (AG) in the parietal cortex, representing regions responsible for storing the memory component of the MUC model (Hagoort, 2013).

The decomposition of the within-frequency electrophysiological vLN into two main spatio-spectrally distinct subnetworks each serving major aspects of language processing is in line with the ubiquitous role of the left IFG in both the ventral and dorsal streams (Saur et al., 2008). Moreover, its central role in the MUC framework (Hagoort, 2005, 2013, 2014) may be underpinned by the wide range of frequencies it operates with, potentially allowing for interactions in different time and spatial scales (Buzsáki and Draguhn, 2004).

### Left Hemisphere Dominant Long-Range Within-Frequency Resting-State Functional Connectivity of the vLN

In contrast to low-level MEG RSNs (e.g., visual, somatosensory and auditory RSNs) disclosing mainly interhemispheric and homotopic rsFC (Brookes et al., 2011; Hipp et al., 2012; Wens et al., 2014a), the neuromagnetic resting-state functional integration from the left IFG within the vLN was essentially left hemisphere dominant. This is in line with the well-established left hemisphere specialization for language concerning around 90% of the general population (for a review see, e.g., Tzourio-Mazoyer et al., 2017). Neuroimaging evidence demonstrates that functional connectivity within the vLN undergoes an age-related developmental shift from prevalently homotopic–interhemispheric in children to mainly left intra-hemispheric functional connectivity in adults (see e.g., Friederici et al., 2011; Perani et al., 2011). The nature of the residual interhemispheric connectivity in adults is hypothesized to be of inhibitory nature, from left to right homotopic regions of the vLN, to maintain the adult left-hemispheric specialization. This would be driven by small-diameter white-matter fibers across higher-order areas (Tzourio-Mazoyer et al., 2017), resulting in the right hemisphere working essentially in an inter-hemispheric manner (Vigneau et al., 2010). The relative lack of right hemisphere neuromagnetic rsFC observed in our study could be related to the higher temporal dispersion caused by these thin non-myelinated callosal fibers (300 ms delays) compared to intrahemispheric pathways (5–10 ms; de Pasquale et al., 2010). Smaller vLN clusters in the right hemisphere compared to those disclosed in previous seed-based fMRI studies (Xiang et al., 2010; Muller and Meyer, 2014) could represent the slower interhemispheric electrophysiological functional integration still captured by slow hemodynamic fluctuations measured by the fMRI technique.

Still, a small cluster composed of two rsFC local maxima located at the right ACC and insula was found in the β and low-γ frequency bands (cluster 3 in Figure 3). Considering their anatomical location, these right hemisphere rsFC local maxima may rather correspond to cross-network interactions of the left IFG rather than to within vLN functional integration.

### The Left IFG Is Involved in Within-Frequency Cross-Network Interactions With the vLN

Cross-network interactions between Broca’s area and nodes of high-level cognitive networks are in line with a domain-general regulatory role of the prefrontal cortex in cognition (Thompson-Schill et al., 2005) and the rich anatomical connectivity of the IFG (Briggs et al., 2019).

First, Broca’s area showed significant neuromagnetic rsFC with the right ACC and insula within β and low-γ bands, respectively. These local correlation maxima were grouped into within-frequency cluster 3 (see Figure 3) and may be part of a goal-directed network (Chang et al., 2013) recruited for cognitively demanding tasks (Seeley et al., 2007). The ACC appears as a key region regulating cognitive and emotional processing (Bush et al., 2000), especially conflict monitoring and modulation of control (Botvinick et al., 2004). The ACC has been shown to exhibit increased engagement in language tasks with high cognitive loads requiring attention, selection and inhibition processes (Piai et al., 2013; Gennari et al., 2018). Given the co-activation of the insula along with the ACC in a wide range of goal-directed tasks (Chang et al., 2013), we hypothesize that the left IFG and the right ACC and insular local correlation maxima belong to a superordinate executive control network (Niendam et al., 2012), which is peripheral to the core vLN and connected, notably, *via* the left IFG, thus consistent with the Control component of the MUC model (Hagoort, 2005, 2013; Hagoort, 2014).

Second, functional integration between the left IFG and bilateral PCC in within-frequency cluster 4 (see Figure 3) was also observed in the α band, corroborating the rsFC between left IFG and PCC previously reported in resting-state fMRI (Muller and Meyer, 2014). In keeping with the role of the DMN, and particularly of the PCC as a core integration hub with other RSNs (de Pasquale et al., 2012; Wens et al., 2019), cross-network interactions have been disclosed between the DMN and the vLN in a study highlighting the Theory of Mind function supported by the DMN (Paunov et al., 2019). Besides, DMN nodes are recruited when updating story representations in narrative language comprehension (Whitney et al., 2009) and are variably influenced by different language tasks (Seghier and Price, 2012). Consistent with the main carrying frequency of the static within-frequency electrophysiological DMN rsFC in the α band (Brookes et al., 2011; Wens et al., 2014b), we propose that within-frequency cluster 4 represents cross vLN-DMN connections specifically integrating left IFG and the PCC.

In sum, our spatial clustering analysis of within-frequency rsFC suggests the existence of two cross-network functional interactions of the vLN mediated by the left IFG with an executive control network (within-frequency cluster 3: right ACC and insula in β and low-γ frequency bands) and postero-medial nodes of the DMN (within-frequency cluster 4: bilateral PCC in the α frequency band).

### The Cross-Frequency Long-Range Resting-State Functional Integration of Broca’s Area

Cross-frequency interactions between distant brain areas have been studied in other contexts with the same envelope correlation metric (e.g., Brookes et al., 2016) or in the context of language with different coupling measurements, such as phase-amplitude coupling (PAC, for a review see, e.g., Benítez-Burraco and Murphy, 2019; and in the particular case of cortical tracking of speech, see e.g., Keitel et al., 2017). Additionally, the prefrontal cortex has been proposed as a recruiter of task-relevant regions in multiple spectral scales to form coherent functional networks (Helfrich and Knight, 2016). As this study is, to the best of our knowledge, the first to investigate cross-frequency power envelope coupling within the vLN, it appears difficult to discuss our results in the light of other types of cross-frequency coupling (e.g., PAC) previously identified during speech processing.

Still, considering the similar spatial signature within the vLN given by both within- (clusters 1 and 2 in Figure 3) and cross-frequency (clusters 4, 5 and 6 in Figure 5) clustering analyses, we propose that genuine cross-frequency functional integration is taking place within the vLN between all four, θ, α, β, and low γ frequency bands (see however the methodological limitation discussed in the next subsection). This hypothesis remains in line with the view that brain functions are simultaneously carried out at multiple temporal and spatial scales (Buzsáki and Draguhn, 2004; Siegel et al., 2012; Lopes da Silva, 2013; Whitman et al., 2013), which is particularly relevant to language comprehension according to Hagoort (2005) and the multi-time resolution processing proposed by Hickok and Poeppel (2007). Contrary to within-frequency electrophysiological vLN coupling, clusters of cross-frequency couplings were not organized spatio-spectrally. Indeed, almost all clusters were composed of cross-frequency rsFC maxima that involved multiple frequency bands. These findings demonstrate that proper investigation of electrophysiological intrinsic vLN rsFC requires the combination of within- and cross-frequency power envelope coupling to fully capture the neural basis of vLN functional integration. They are also in agreement with the *multiplexed neural coding* theory about the functional role of networks oscillations (Akam and Kullmann, 2014). Multiplexing is the process of combining multiple signals for transmission through a single communication channel, in such as way that distinct components can be independently recovered from the transmitted signal (Akam and Kullmann, 2014). We hypothesize that, similarly to task-based studies that narrowed down specific language processes in specific frequency bands and regions (e.g., Ihara et al., 2003; Bastiaansen et al., 2005; Gehrig et al., 2012; Obleser and Weisz, 2012; Liljeström et al., 2015; Rommers et al., 2017; Wang et al., 2018), the within-frequency neuromagnetic vLN is segmented in functionally and spectro-spatially specific ventro-dorsal subnetworks, while, conversely, spatial patterns of cross-frequency vLN functional integration are spectrally more widespread and involve multiple frequency bands. These different modes of vLN functional integration may serve to create channels or routes for selective communication among the different vLN streams, and between the vLN and other high-level cognitive networks.

Of note, cross-frequency cluster 7 (Figure 5), encompassing right temporo-parietal regions that may represent the right aspect of the bilateral ventral vLN (Hickok and Poeppel, 2007), was more restricted to specific cross-frequency coupling interactions. This cluster was indeed defined by low cross-frequency (θ-to-α and α-to-θ) functional integration (notwithstanding the methodological limitation discussed in the next subsection) and therefore in line with the task-based literature (Bastiaansen et al., 2005; Obleser and Weisz, 2012; Rommers et al., 2017; Wang et al., 2018), and the contralateral within-frequency cluster 1 (Figure 3) carried in θ and α bands. This may reflect a different time scale or less direct functional integration of the right ventral vLN.

### Methodological Considerations

The seed-based correlation analysis used here, despite having proven successful to reproduce other fMRI RSNs in MEG using seeds from the fMRI literature (Brookes et al., 2012; Hipp et al., 2012; Wens et al., 2014b), is relatively more fraught with signal leakage (between voxels in close proximity to the seed) compared to data-driven independent component analysis (Brookes et al., 2011). Static orthogonalization was therefore used for spatial leakage correction (Brookes et al., 2012), as it is moderately conservative and relatively resilient to seed mislocation (Wens et al., 2015). Using this approach, we were able to identify, in the θ and α frequency bands, long-range connections from the left IFG to left temporo-parietal vLN regions that are spatially substantially distant from the seed and for which the involvement of spurious functional connectivity after leakage correction can be ruled out (Wens et al., 2015; Palva et al., 2018). By contrast, in the β and low γ frequency bands, connections were closer to the left IFG seed, albeit with the emergence of clearly separate correlation local maxima in key nodes of the vLN, such as the left opercular or insular regions. Still, considering the relatively close proximity between these brain areas, the influence of residual spurious functional connectivity after leakage correction cannot be totally excluded, although intracranial recordings have clearly demonstrated the transfer of information between Broca’s area and motor frontal regions during speech production (Flinker et al., 2015).

To infer the functional relevance of our neuromagnetic rsFC local maxima relative to the vLN, we relied on a meta-analytic vLN template issued from the Neurosynth online platform (Yarkoni et al., 2011) based on 885 fMRI studies. A local rsFC maximum was deemed to fall within the vLN template when its distance to the mask (i.e., the Euclidean distance to the closest in-mask voxel) was less than 10 mm. Still, the establishment of the fMRI-MEG relationship was somehow limited by the potential biases in the automated generation of the meta-analytic vLN template (e.g., heterogeneous studies and populations, sporadic MNI coordinate extraction errors—for more details, see Yarkoni et al., 2011), and the spatial tolerance threshold of 10 mm needed to account for systematic spatial differences between fMRI and MEG. Of note, this threshold was kept below the average inter-modality difference reported in the corresponding articles (e.g., 15 mm in Liljeström et al., 2009).

We chose to focus on cortical parcels of the AAL atlas to avoid potential deep artifactual local rsFC maxima. This approach precluded the inclusion of relevant vLN regions such as deep nuclei and cerebellar hemispheres (see e.g., Price, 2012), for which the ability of MEG to detect proper neural activity is still a matter of debate.

Finally, some of our findings about cross-frequency rsFC, specifically interactions between neighboring frequency bands, should be considered cautiously. They may represent physiological interactions between dynamics genuinely taking place at different frequencies, but they also might merely reflect within-frequency coupling spectrally leaking from the main carrying frequency band. In this regard, the several rsFC maxima disclosed between theta-band F3tv and posterior parietal low-gamma band activity (Figure 4, top row) provides our most salient example of genuine cross-frequency coupling within the neuromagnetic vLN.

### Conclusion

Contrary to other neuromagnetic RSNs shown to be driven by one main carrying frequency, this MEG study demonstrates that four different carrying frequency bands and their cross-frequency interactions drive the rsFC from the left IFG along the perisylvian vLN. Importantly, the within-frequency vLN rsFC is characterized by a spatio-spectral pattern consistent with the dual stream model. Refining the internal architecture of the resting-state vLN previously uncovered in fMRI as a whole, this MEG study identifies a ventro-caudal subnetwork comprising left temporo-parietal regions carried mainly by the θ and α frequency bands, segregated from a dorso-rostral subnetwork of left fronto-insular regions carried by the β and low-γ frequency bands. In contrast, the cross-frequency vLN rsFC discloses spatial patterns of cross-frequency vLN functional integration that are spectrally more widespread and involve multiple frequency bands. These different modes of vLN network oscillations are in agreement with the multiplexed neural coding hypothesis at the basis of selective communication within and between brain networks.

Moreover, the static intrinsic functional architecture of the neuromagnetic human vLN, both intra- and cross-frequency, involves clearly left hemisphere dominant vLN integration as well as cross-network interactions with the executive control network and postero-medial nodes of the DMN.

Finally, this study is in line with the ubiquitous role of the left IFG in the dual stream model and confirms its central role in the MUC framework in the perspective of a multi-frequency and spatially distinct long-range functional integration with other regions of the vLN and other functional networks.

Overall, this study lays the foundations for the investigation of the alterations in electrophysiological intrinsic vLN functional integration induced by language-related brain disorders with a method free of any neurovascular coupling bias such as fMRI.

## Data Availability Statement

The datasets generated for this study are available on request to the corresponding author.

## Ethics Statement

The studies involving human participants were reviewed and approved by CUB—Hôpital Erasme Ethics Committee. The patients/participants provided their written informed consent to participate in this study.

## Author Contributions

TC, VW, and XDT conceived and designed the study. MV, AM, and MB acquired the data. TC and VW analyzed the data. TC, VW, and XDT wrote the manuscript. VW implemented the methods, with input from MB. GN, PP, NS, and SG took part in the interpretation of the data and provided substantial input for the manuscript. All authors contributed to manuscript revision, read and approved the submitted version.

## Conflict of Interest

The authors declare that the research was conducted in the absence of any commercial or financial relationships that could be construed as a potential conflict of interest.
